# Proteomic analysis of the organic matrix of the abalone *Haliotis asinina *calcified shell

**DOI:** 10.1186/1477-5956-8-54

**Published:** 2010-11-04

**Authors:** Benjamin Marie, Arul Marie, Daniel J Jackson, Lionel Dubost, Bernard M Degnan, Christian Milet, Frédéric Marin

**Affiliations:** 1 UMR 5561 CNRS, Biogéosciences, Université de Bourgogne, 21000 Dijon, France; 2 Département RDDM, Plateforme de Spectrométrie de Masse et de Protéomique/FRE3206 CNRS, Molécules de Communication et Adaptations des Micro-organismes, M.N.H.N, 75005 Paris, France; 3 Courant Research Center Geobiology, Georg-August-University of Göttingen, 37077 Göttingen, Germany; 4 School of Biological Sciences, University of Queensland, 4072 Queensland, Australia; 5 UMR 7208 BOREA, M.N.H.N., 75005 Paris, France

## Abstract

**Background:**

The formation of the molluscan shell is regulated to a large extent by a matrix of extracellular macromolecules that are secreted by the shell forming tissue, the mantle. This so called "calcifying matrix" is a complex mixture of proteins and glycoproteins that is assembled and occluded within the mineral phase during the calcification process. While the importance of the calcifying matrix to shell formation has long been appreciated, most of its protein components remain uncharacterised.

**Results:**

Recent expressed sequence tag (EST) investigations of the mantle tissue from the tropical abalone (*Haliotis asinina*) provide an opportunity to further characterise the proteins in the shell by a proteomic approach. In this study, we have identified a total of 14 proteins from distinct calcified layers of the shell. Only two of these proteins have been previously characterised from abalone shells. Among the novel proteins are several glutamine- and methionine-rich motifs and hydrophobic glycine-, alanine- and acidic aspartate-rich domains. In addition, two of the new proteins contained Kunitz-like and WAP (whey acidic protein) protease inhibitor domains.

**Conclusion:**

This is one of the first comprehensive proteomic study of a molluscan shell, and should provide a platform for further characterization of matrix protein functions and interactions.

## Background

The calcified molluscan shell is an excellent model with which to study the process of biomineral formation. The wide morphological diversity of shell-bearing molluscs (bivalves, gastropods, cephalopods, monoplacophorans and scaphopods) also extends to a tremendous diversity of shell micro-textures. Despite this diversity, molluscan shells are produced by an evolutionarily homologous structure known as the mantle. The polymorph of CaCO_3 _(primarily aragonite or calcite), along with all other nano-scale features of the biomineral, are thought to be determined and regulated by an extracellular 'cell-free' matrix that is secreted by the mantle. This matrix is incorporated into and surrounds nascent CaCO_3 _crystals during shell growth. Even though it constitutes only a small part of the total shell weight (1-5%), this matrix is clearly essential for initiating biomineral formation and imparting critical physical properties to the shell such as fracture resistance. The biochemical characteristics of the matrix, usually purified and studied following decalcification of the shell, indicate that it is comprised of a heterogenous set of macromolecules including chitin, hydrophobic 'framework' proteins and soluble proteins and glycoproteins [1]. However, relatively few matrix proteins have been identified and characterised from abalone shells, perhaps the best-studied gastropod biomineralisation system. To date these include Lustrin-A [2], Perlucin [3], Perlustrin [4], AP7, AP24 [5], Perlwapin [6] and Perlinhibin [7].

Jackson et al. [8-10] employed a high-throughput EST sequencing strategies in order to identify gene products that may be directly involved in shell formation of the tropical abalone *Haliotis asinina*. Hundreds of conceptually translated proteins putatively related to shell calcification were identified using this approach, however none of these have been directly characterized from the shell. Furthermore, this approach cannot accurately discriminate between proteins required for non-mineralising functions, and those directly involved in shell formation. When coupled with a proteomic approach such as we have employed here, these EST libraries constitute a valuable resource for the accurate identification and annotation of potentially full length, true shell forming proteins. Here we have been able to unambiguously annotate 14 proteins from the nacreous (inner/ventral most) and prismatic (outer/dorsal most) shell layers of *H. asinina*. One of these proteins, *Has*-Perlwapin, is homologous to a protein previously described from the nacre of *Haliotis laevigata *[6]. We also identified *Has*-Sometsuke which is thought to be involved in pigmenting the outer periostracum of *H. asinina *[8,9] but apparently plays other shell forming roles, while *Has*CL10contig2 was identified from a mantle EST study but was not characterised in anyway [10]. All 11 other shell proteins are novel and most do not exhibit any homology with proteins from public databases.

Given the high proportion of novel genes being reported from non-model EST datasets and the growing flood of sequence data from next generation technologies, these results emphasize the importance of proteomic approaches for the validation of coding sequences. This is especially relevant for the field of molluscan biomineralization where most of the characterised biomineral-associated proteins have no known homologs in any model species [11].

## Methods

### Shell matrix extraction

Fresh *Haliotis asinina *shells (10-12 cm in length) were collected from the Bribie Island Aquaculture Research Facility (Queensland, Australia). Superficial organic contaminants as well as the periostracum were removed by incubating intact shells in NaOCl (1%, v/v) for 24 h. Shells were then thoroughly rinsed with water and then roughly crushed into approximately 1-mm^2 ^fragments, and subsequently into fine powder (>200 μm). For some shells, the external prismatic layer was removed by abrasion under cold water to avoid shell heating, allowing the proteinaceous components of the nacreous layer alone to be extracted. All protein extractions were performed at 4°C as previously described [12]. Briefly, powdered samples (nacre only or nacre + prisms) were decalcified overnight in cold dilute acetic acid (5%, v/v), which was slowly added by an automated titrator (Titronic Universal, Schott, Mainz, Germany) at a flow rate of 100 μL every 5 s. The solution (final pH around 4.2) was centrifuged at 3,900 g (30 min). The resulting pellet, corresponding to the acid-insoluble matrix (AIM), was rinsed 6 times with MilliQ water, freeze-dried and weighed. The supernatant containing acetic acid-soluble matrix (ASM) was filtered (5 μm) and concentrated with an Amicon ultra-filtration system on Millipore^® ^membrane (YM10; 10 kDa cut-off). The solution (about 5-10 mL) was extensively dialyzed against 1 L of MilliQ water (over at least three days with 6 water changes) before being freeze-dried and weighed.

### Sample preparation for proteomic analysis

Following SDS-PAGE under denaturing conditions (4-15% acrylamide gel) and staining with Coomassie Brilliant Blue (CBB), bands selected for further investigation were excised and completely destained by 3 washes in 200 μL of a 50/50 mixture of 100 mM NH_4_HCO_3 _pH 8.1 and 100% acetonitrile (ACN) for 30 min at 37°C. Reduction was performed with 100 μL of 20 mM dithiothreitol (DTT) in 50 mM NH_4_HCO_3 _pH 8.1 (30 min at 37°C) after which the supernatant was removed. Alkylation was performed with 200 μL of 50 mM iodoacetamide in 50 mM NH_4_HCO_3 _pH 8.1 for 30 min at room temperature in the dark. The supernatant was then removed and gel slices were rinsed with 300 μL of 25 mM NH_4_HCO_3 _pH 8.1, then with 300 μL of 100% ACN and dried under vacuum. Gel slices were treated with 1 μg of trypsin (T6567, proteomics grade, Bio-Rad) in 100 μL of 20 mM NH_4_HCO_3 _pH 8.1 with 10% ACN for 8 h at 37°C. The supernatant was then collected and freeze dried. Samples were re-suspended in 20 μL of 0.1% TFA, and 5 μL was injected into the nanoLC-ESI-MS/MS system for analysis. This in gel digestion procedure was performed for 12 and 8 protein bands derived from nacre + prisms AIM and ASM extracts, respectively.

In solution digestions of ASM and AIM from nacre only and nacre + prism samples were also performed. In each case, one mg of organic matrix material was reduced with 100 μL of 10 mM DTT (Sigma-Aldrich, France) in 100 mM NH_4_HCO_3 _pH 8.1 for 30 min at 57°C. Fifteen μL of iodoacetamine (50 mM, final concentration) was added and alkylation was performed for 30 min at room temperature in the dark. Samples were then freeze dried and re-suspended in 200 μL of a solution containing 5 μg of trypsin (T6567, proteomics grade, Bio-Rad) in 50 mM NH_4_HCO_3 _pH 8.1 with 5% ACN, and then incubated overnight at 37°C. Samples were centrifuged (30 min, 14,000 g) and the supernatants transferred to new tubes before being freeze dried and re-suspended in 100 μL of 0.1% TFA. Five μL of each sample was injected into the nanoLC-ESI-MS/MS system analysis.

### Peptide fractionation and data acquisition

High performance liquid chromatography (HPLC) of the tryptic peptides was performed on a C_18 _column (Interchim, 1 mm × 150 mm, 5 μm, 300°A) at a flow rate of 50 μL.min^-1 ^with a linear gradient (5 to 80% in 90 min) of acetonitrile and 0.1% formic acid. The fractionated peptides were analyzed with an electrospray ionization quadripole time-of-flight (ESI-QqTOF) hybrid mass spectrometer (pulsar i, Applied Biosystems) using information dependent acquisition (IDA), which allows switching between MS and MS/MS experiments. The data were acquired and analyzed with the Analyst QS software (Version1.1). After 1 s acquisition of the MS spectrum, the two most intense multiple charged precursor ions (+2 to +4) could be selected for 2 s-MS/MS spectral acquisitions. The mass-to-charge ratios of the precursor ions selected were excluded for 60 s to avoid re-analysis. The minimum threshold intensity of the ion was set to 10 counts. The ion-spray potential and declustering potential were 5200 V and 50 V, respectively. The collision energy for the gas phase fragmentation of the precursor ions were determined automatically by the IDA based on their mass-to-charge ratio (*m/z*) values.

### Data analysis

The MS/MS data were used for database searches using an in house version of the MASCOT search engine (Matrix Science, London, UK; version 2.1). 8,335 EST and 832 nucleotide sequences derived from *H. asinina *EST libraries were downloaded (January 2010) from the NCBI server (http://www.ncbi.nlm.nih.gov) and MASCOT searches were directly performed against nucleotide sequences. LC-MS/MS data generated by each shell sample and protein band were searched separately, using carbamido-methylation as a fixed modification and methionine oxidation as variable modification. The peptide MS tolerance was set to 0.5 Da and the MS/MS tolerance was set to 0.5 Da. The threshold score for peptide identification was set between 28 and 31 for each search. *In silico *translated nucleotide sequences with at least two independent peptide matches were considered to be valid. All peptide hits were manually confirmed by the interpretation of the raw LC-MS/MS spectra with analyst QS software (Version 1.1). Quality criteria were the peptide MS value, the assignment of major peaks to uninterrupted y- and b-ion series of at least 3-4 consecutive amino acids and the match with the *de novo *interpretations proposed by the software.

Protein identification was attempted using BLAST searches against the UniProtKB/Swiss-Prot protein sequence database (http://www.uniprot.org) and the GenBank non-redundant (nr) database (http://www.ncbi.nlm.nih.gov/blast.cgi). To detect sequences sharing similarity with *H. asinina *shell proteins from other molluscan EST projects (which are not deposited in GenBank nr), tBLASTn searches was also performed against GenBank dbEST and were restricted to molluscan taxa (taxid:6447). Signal peptides were predicted using SignalP 3.0 (http://www.cbs.dtu.dk/services/SignalP) and conserved domains were predicted using SMART (http://smart.embl-heidelberg.de). Following peptide signal removal theoretical masses and p*I*s were determined using the EXPASY PROTPARAM tool (http://www.expasy.org/tools/protparam.html). Alignments were performed with Clustal-W or hierarchical-clustering algorithms using UniProt (http://www.uniprot.org) or the MULTALIN (http://bioinfo.genotoul.fr/multalin/multalin.html) online tools.

## Results and Discussion

**Figure 1:**
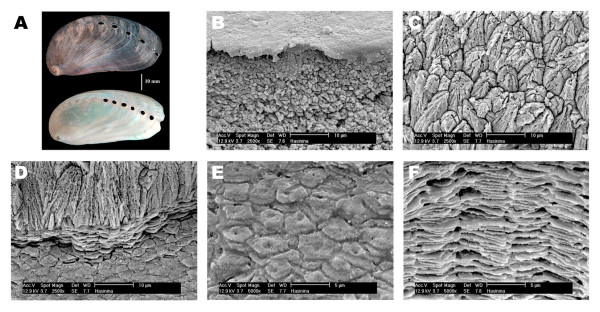
**The shell layers of the tropical abalone of *Haliotis asinina***. (A) General view of the shell before (top) and after (lower) the mechanical removal of the periostracal and prismatic layers. (B) SEM micrograph of a cross-section through the abalone shell illustrating details of the periostracum (top) and of the calcified prismatic layer (lower). (C) SEM micrograph illustrating details of the prismatic layer. (D) SEM micrograph of the boundary between the prismatic (top) and the nacreous layer (lower). (E) SEM micrograph of the nacreous layer (oblique view). (F) SEM micrograph of the nacreous layer (cross section) illustrating the columnar superimposition of nacre tablets that is characteristic of gastropod nacre.

Like other haliotid gastropods, *H. asinina *exhibits a multi-layered shell (Figure 1). The thin external periostracum is primarily composed of organic components and is not calcified (Figure 1A-B). The underlying layers are highly calcified and consist of a fine outer prismatic layer and a thick inner nacreous layer. Prisms are micro-needles enveloped by an organic sheath (Figure 1B-D). Nacre consists of the columnar superimposition of 0.5 μm thick aragonitic tablets, embedded within a peripheral thin organic matrix (Figure 1D-F). By carefully removing the periostracum with sodium hypochlorite, we were able to subsequently extract the matrix associated with both prismatic and nacreous calcified layers, or with the nacreous layer alone (Figure 1A). Of the nacre + prism material the AIM represents around 1.5% by weight, and the ASM 0.05-0.1%.

**Figure 2:**
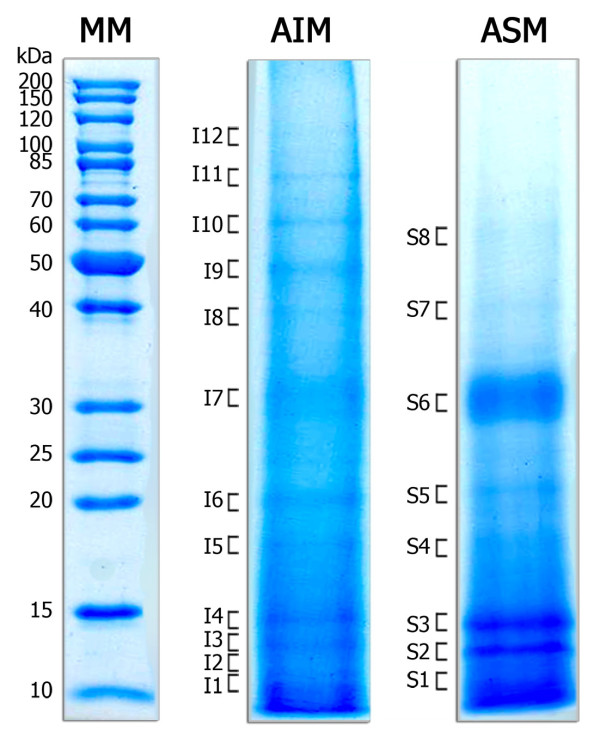
**SDS-PAGE fractionation of acid-soluble (ASM) and acid-insoluble (AIM) shell matrix proteins**. The mass of molecular weight markers (in kDa) is indicated on the left. Following electrophoresis under denaturing condition, proteins were stained with CBB. Approximately 80 μg of protein material were applied per well. I1-I12 and S1-S8 correspond to bands that were excised for in-gel digestion and MS/MS analysis.

When analysed by one-dimensional electrophoresis under denaturing conditions, the nacre + prism ASM and AIM displayed few discrete prominent bands (Figure 2). When present, patterns of ASM and AIM staining appear to share similar bands, such as the thick band at 32 kDa and the thin discrete bands migrating at between 15 and 10 kDa. Twelve and eight gel bands were collected from nacre + prism AIM and ASM extractions respectively, and were processed as described above for LC-MS/MS analysis. Un-fractionated ASM and AIM material derived from the nacreous layer alone and the nacre + prism samples were similarly analyzed by LC-MS/MS. These analyses were performed three times for the un-fractionated matrices, and once or twice for the SDS-PAGE bands. For all samples, the peak list generated from the MS/MS spectra was directly interrogated against the *H. asinina *EST database using MASCOT software. In this way we were able to identify 14 proteins from the EST data (Table 1 and additional file 1, Table S1). The putative identifications of three other proteins, based on only one unique peptide, are indicated in additional file 2, Table S2, but are not discussed further here. No additional peptides were identified by including phosphorylation as a variable modification during the MASCOT searches, indicating that specific enrichment and LC-MS procedures are needed to analyze these post-translational modifications. Nevertheless, 3 of the identified proteins - ML5A7, ML1E6 and ML5H8 - were mostly detected in the upper regions of the SDS-PAGE gel. The discrepancy between their position in the gel and their lower theoretical molecular weight suggests either that the mature form of these proteins exhibits post-translational modifications, and/or that the full-length cDNA is not represented in the EST library.

**Table 1:** Pairing and annotating *Haliotis asinina *ESTs with shell derived MS/MS peptides

Protein name [GenBank Accession]	EST library*	Homology/ domain	Mass kDa (Observed/ Theoretical)	Complete sequence/ SignalP	Source of material			Best MASCOT protein scores	Number of matching peptides
					Band ID	Nacre	Pr + Nc		
ML5A7 [DW986289]	1, 2	No homology/no recognised domains	32/25	Yes/Yes	S6 I7	- -	ASM AIM	459	9
HasCL10contig2 [EZ420619]	3	Fibroin-like/Gly and Ala rich domains	-/44	Yes/Yes	- -	ASM AIM	ASM AIM	455	7
P0012N13_463 [GT274423]	3	Papilin/2 Kunitz domains	14/≥15	No/No	S3 I4	ASM AIM	ASM AIM	362	6
ML1E6 [DW986219]	1, 2	Sometsuke/ependymin domain	32/20	Yes/Yes	S6 I7	- -	ASM AIM	347	7
P0006O07_675 [GT272916]	3	No homology/no recognised domains	13-48/≥19	No/No	S2-S7 I3-I9	ASM AIM	ASM AIM	343	6
P0025F23_658 [GT276990]	3	No homology/Asp and Ala rich domains	32/≥23	No/No	- I7	- AIM	- AIM	343	3
ML3A11 [DW986237]	1, 2, 3	No homology/Pro rich domain	15/16	Yes/Yes	S3 I4	ASM AIM	ASM AIM	336	4
ML6A10 [DW986342]	1, 2, 3	No homology/no recognised domains	13/12	Yes/Yes	S2 I3	ASM AIM	ASM AIM	320	5
ML8B1 [DW986463]	1, 2, 3	No homology/Gln rich domains	13/11	Yes/Yes	S2 I3	ASM AIM	ASM AIM	195	3
P0011O14_517 [GT274178]	3	No homology/no recognised domains	18/≥16	No/Yes	- I5	- AIM	- AIM	189	3
6G3 [GD272908]	2	No homology/ependymin domain	21/21	Yes/Yes	S5 I6	ASM AIM	ASM AIM	173	3
ML3E9 [DW986256]	1,2, 3	Perlwapin/3 WAP domains	-/≥15	No/No	- -	- -	ASM AIM	148	3
ML5B8 [DW986296]	1, 2	No homology/no recognised domains	32/21	Yes/Yes	S6 -	- -	ASM	125	2
ML5H8 [DW986339]	1, 2, 3	No homology/Chitin-Binding domain	-/10	Yes/Yes	- -	- -	ASM AIM	116	3

*EST libraries: 1 = *H. asinina *juvenile mantle SMART cDNA library [8]; 2 = *H. asinina *developmental microarray library [9]; 3 = *H. asinina *adulte mantle cDNA library [10]. "Pr + Nc" indicates "Prisms + nacre" extract. "≥" indicates that the theoretical molecular mass is at least the following value, as some sequence information is missing.

Interestingly most of the 14 proteins which matches *H. asinina *ESTs are present in both the ASM and AIM, a finding that is in agreement with previous observations [13,14]. This might be explained by successive protein maturation events such as progressive insolubilization via cross-linking and/or protein tanning. The organic matrix derived from nacre material alone contains at least 9 of these 14 proteins. In contrast, conceptually derived proteins ML5A7, ML1E6 (*Has*-Sometsuke), ML3E9, ML5B8 and ML5H8 proteins were detected in the nacre + prism material, but not in the nacre alone, suggesting that they are restricted to the prismatic layer. We propose that these proteins are involved in regulating the growth and/or orientation of the prismatic layer, however further *in vitro *tests should be performed to test this hypothesis. Even though we carefully removed the periostracum from the external shell surface by chemical treatment, we also cannot exclude the possibility that these latter proteins are also constituents of the periostracal layer. We also found that all conceptually translated EST sequences that match our MS/MS peptides possess a signal peptide, indicating that these bioinformatically predicted proteins are likely to represent the entire amino N-terminus and are genuinely secreted by the mantle epithelium.

### New Gln- and Met-rich proteins

**Figure 3:**
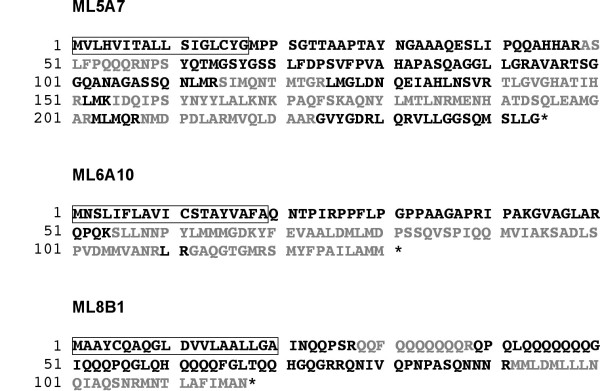
**Sequences of ML5A7, ML6A10 and ML8B1, 3 new proteins with Gln- and Met-rich domains**. Predicted signal peptides are boxed. Peptides identified by MS/MS are in grey. An asterisk indicate the stop codon.

Three of the 14 proteins that we have identified here do not exhibit any sequence similarity with any other proteins, and contain unusually rich Met and Gln domains (Figure 3; Additional file 1, Table S1). Putative full-length ORFs for these 3 proteins were deduced from *H. asinina *ESTs ML5A7, ML8B1 and ML6A10. Peptides matching ML5A7 were only detected in nacre + prismatic samples. Conversely peptides matching ML8B1 and ML6A10 were detected in nacre only. Following signal sequence removal ML5A7, ML8B1 and ML6A10 are characterized by theoretical p*I*s between 10 and 12, and theoretical molecular weights of 25, 11 and 12 kDa, respectively. The protein encoded by ML5A7 is enriched in Ala (13%) and Leu (10%), while the product of ML6A10 is enriched in Ala (13%), Pro (13%) and Met (11%). ML8B1 encodes a protein with a remarkable Gln content (38%), corresponding to three short poly-glutamine motifs located in the N-terminus. All three protein sequences are also remarkably methionine rich (7-11%). The occurrence of such Met- and Gln-rich domains is an uncommon feature of biomineralizing proteins, and we suggest that their occurrence may be related to specific functions: previous *in vitro *experiments have shown that poly-glutamine domains are responsible for protein aggregation [15], whereas poly-methionine domains stabilize the precipitation of calcium carbonate crystals [16]. Functional characterization of these proteins by *in vitro *calcification assays are needed to test these hypotheses.

### Repetitive low complexity domain (RLCD) containing proteins

Several mollusc-shell proteins are known to exhibit either repetitive motifs, or domains of low complexity. For example, Nacrein contains a large GN-domain [17], MSI60 exhibits 39 poly-G blocks and 11 poly-A blocks [18] and MSP-1 contains GS domains that alternate with D-rich domains [19]. It has been proposed that acidic repetitive low complexity domain proteins (RLCDs) might be a prominent component of the matrix framework [1,18]. More recently, Suzuki and co-workers [20] hypothesized that the acidic poly-D blocks within Pif-80 binds *in vitro *to calcium carbonate crystals. Because alignment-based sequence comparisons between proteins that contain extensive low-complexity regions are often phylogenetically uninformative and can lead to false positive results, phylogenetic analyses are difficult to perform using these protein sequences [21]. However, it is likely that RLCD containing proteins are important constituents of the biomineralization "toolkit".

**Figure 4:**
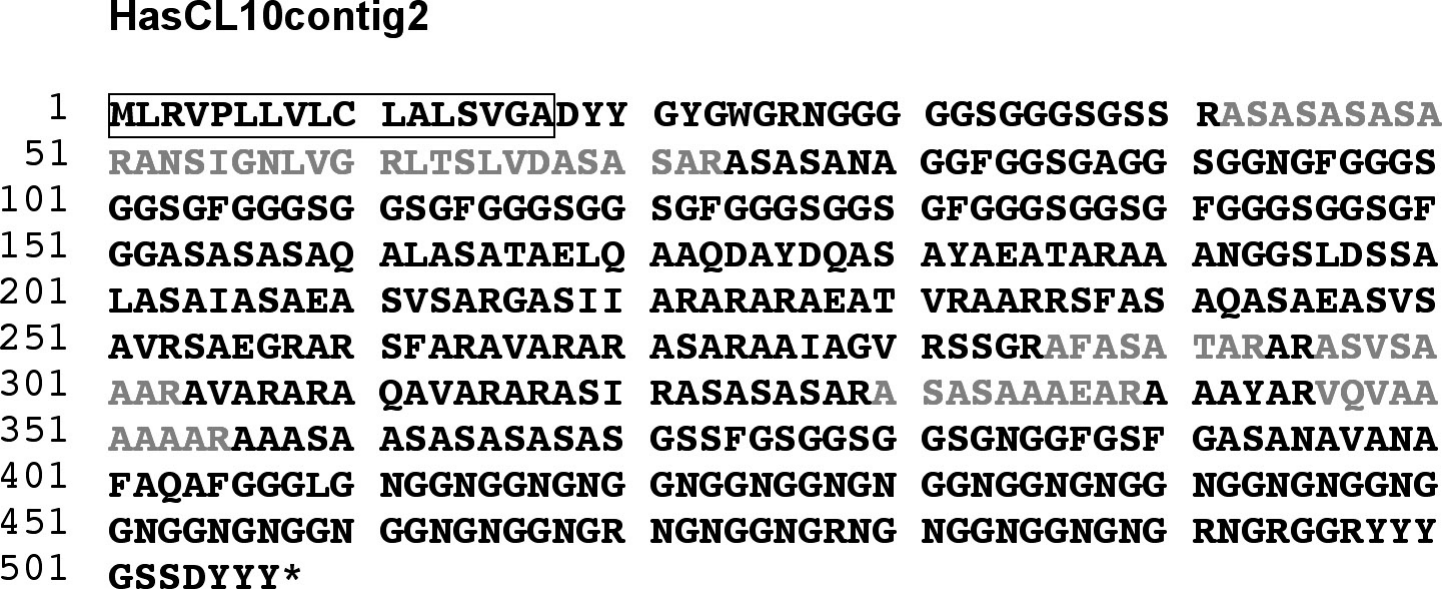
**Sequence of HasCL10contig2, a repetitive low complexity domain (RLCD) protein**. The predicted signal peptide is boxed. Peptides identified by MS/MS are in grey. An asterisk indicates the stop codon.

We have identified proteins with (RLCDs) from the shell of *H. asinina*. In particular, HasCL10contig2 (Figure 4) and P0025F23_658 (Additional file 1, Table S1) transcripts encode fibroin-like proteins, which possess low complexity hydrophobic or acidic domains. HasCL10contig2 is highly expressed in the mantle [10] and encodes a 507 amino acid long protein with a 17-amino acid signal peptide (Figure 4). When the signal peptide is removed the predicted protein exhibits a theoretical molecular mass of 44 kDa and a calculated p*I *around 12. Peptides matching this protein are largely present in both the ASM and AIM extracted from nacre without SDS-PAGE fractionation. Interestingly, the sequence of the HasCL10contig2 protein contains numerous RLCDs and hydrophobic domains including 7 collagen-like -GGSGGxGFG- repeats, 26 -GNG- repeats and A-rich blocks with high amount of Ser (S) and Lys (R), that potentially make it poorly suitable for SDS-PAGE separation.

### Protease inhibitor proteins

**Figure 5:**
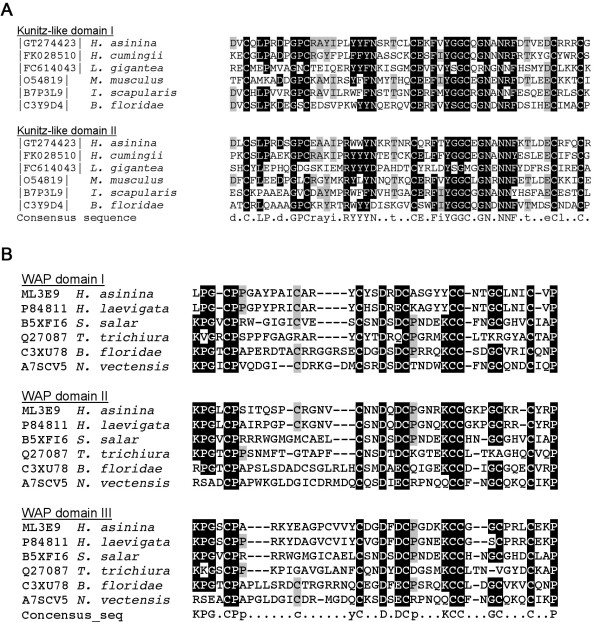
**Sequences of 2 protease inhibitor proteins**. These protein sequences are deduced from translated sequences of the entry P0012N13_463 (A) and ML3E9 (B). (A) An alignment of the 2 Kunitz domains with other protease inhibitor proteins from diverse mollusc and metazoan origins indicates that the Kunitz-like protein family is highly conserved. (B) An alignment of the 3 WAP domains of *Has*-Perlwapin with other molluscan and metazoan Perlwapin proteins reveals the high degree of conservation of the cysteine residues. Positions shaded in black indicate cases where more than 70% of the residues are identical, and grey where at least 40% of the residues are identical or share biochemical similarity with the consensus residue. GenBank or Swiss-Prot numbers are indicated on the left.

Two protease inhibitor domain-containing proteins were identified in the shell matrix of *H. asinina *(Figure 5; Additional file 1, Table S1). MS/MS peptides matching *H. asinina *EST P0012N13_463 encode a protein that contains 2 consecutive Kunitz-like protease inhibitor domains. MS/MS peptides also matched EST ML3E9 that corresponds to a homolog of Perlwapin, which has been previously described from the nacre of *Haliotis laevigata *[6]. Perlwapin exhibits 3 successive WAP (Whey Acidic Protein) domains. Sequence alignments with other Kunitz-like and WAP domain containing proteins from diverse molluscs and metazoans (Figure 5A and 5B, respectively) show that these two protein families are highly conserved across the Metazoa.

The presence of protease inhibitors in an acellular biomineral is at first puzzling, however this is not an isolated obervation. In the abalones *Haliotis rufescens *and *Haliotis laevigata*, two nacre proteins, Lustrin-A [2] and Perlwapin [6], contain WAP protease inhibitor-like domains. Recently, Bédouet and co-workers [22] demonstrated the presence of active cysteine-proteinase inhibitors in the nacre matrix of the pearl oyster *Pinctada margaritifera*. Furthermore, Liu and co-workers described a putative secreted protein from *Pinctada fucata *mantle cells that exhibits sequence homology with Kunitz-like and WAP domains [23]. Similar findings were published for other non-molluscan biocalcifying models: a Kazal-type serine-protease inhibitor has recently been described from the sea urchin calcified-skeleton matrix [24,25], and a recent proteomic analysis has demonstrated the presence of numerous protease inhibitors from the chicken egg shell [13]. Protease inhibitors constitute a wide group of ubiquitous proteins that are involved in many biological functions. Our data suggests that the presence of protease inhibitor domains in biomineral associated proteins play roles (as yet undefined) for calcified biomineral formation and/or maintenance. We postulate that the protection of the organic matrix against degradation by exopeptidases is the most likely function of these protease inhibitors. Alternative functions may include roles in remodelling the shell matrix or in the regulation (activation or inactivation) of other multi-domain matrix components [26].

### Ependymin related proteins

**Figure 6:**
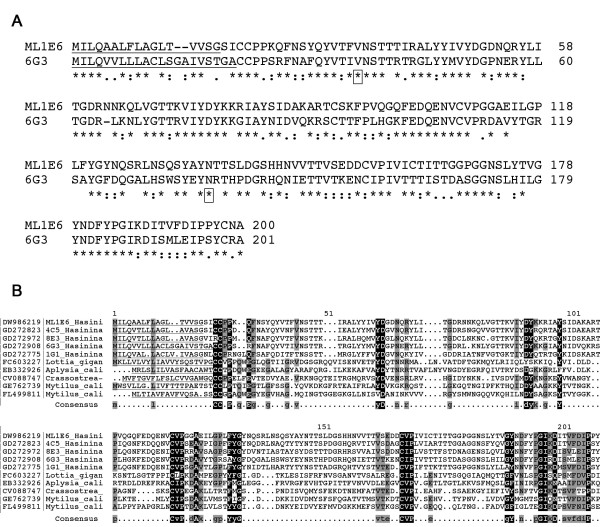
**Sequences of ML1E6 and 6G3, 2 ependymin-related proteins**. (A) An alignment of 2 shell matrix ependymin-related proteins. Putative N-glycosylation sites are boxed. Predicted signal peptides are underlined. (B) An alignment of ML1E6 and 6G3 of *H. asinina *with other mollusc ependymin-related proteins reveals the high degree of conservation of the cysteine residues between the different forms. Positions shaded in black indicate cases where more than 70% of the residues are identical, and grey where at least 40% of the residues are identical or share biochemical similarity with the consensus residue. GenBank or Swiss-Prot numbers are indicated on the left.

Two different ependymin-related proteins, ML1E6 and 6G3, were observed in the shell matrices of *Haliotis asinina *(Figure 6; Additional file 1, Table S1). The ML1E6 transcript, *Has-sometsuke*, is expressed in the anterior zone of the outer fold of the mantle and maps precisely to patterns of shell pigmentation, the protein product of which is therefore very likely located in the periostracum [8]. Interestingly, we find the *Has*-Sometsuke protein in the external prismatic shell layer and is very likely also present in the periostracal layer, whereas 6G3, another ependymin-related protein, is also present in the nacreous layer. Ependymin-related proteins constitute a family of extracellular glycoproteins of about 200 amino acids containing 6 cysteine residues, four of which are highly conserved and probably form intramolecular disulfide bonds, and two putative N-linked glycosylation sites [27] (Figure 6A). Furthermore, we notice that ML1E6 was mainly detected on SDS-PAGE in a 32-kDa band whereas its theoretical mass is 20 kDa, suggesting that *Has*-Sometsuke is post-translationally modified, probably by glycosylation. *Has*-sometsuke has a theoretical p*I *of 5.6 and is the only acidic protein we observed from our shell matrix analysis. BLAST alignments indicate that ML1E6 and 6G3 exhibit similarity with translated ESTs derived from four molluscs (Figure 6B), but weaker similarity to deuterostome ependymins [8,9]. Various functional features of this protein family, including its ability to bind calcium via N-linked sialic acids residues [28] and to undergo polymerization into insoluble fibrils [29] support the hypothesis that they play a direct role in the organization of the shell-matrix framework.

### Putative carbohydrate-binding protein

**Figure 7:**
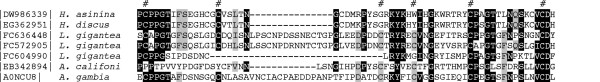
**Sequence of ML5H8, a novel putative chitin-binding protein**. An alignment of the ML5H8 protein with the sequences of chitin-binding domains of peritrophin-A type from molluscs and arthropods. Positions with < 80% and < 60% conservation are indicated in black and gray, respectively. Peritrophin-A is an extracellular domain that contains six conserved cysteines (indicated by #) that probably form three disulphide bridges. GenBank or Swiss-Prot numbers are indicated on the left.

Our MS/MS peptides also map to an *H. asinina *EST (ML5H8), which codes for a secreted protein that exhibits sequence similarity with carbohydrate-interacting proteins (Figure 7; Additional file 1, Table S1). The conceptually derived mature protein sequence of ML5H8 exhibits a calculated mass of 10 kDa, and a p*I *of 10. ML5H8 contains a short domain rich in cysteine that shows similarity to the chitin-binding domain of peritrophin-A (Pfam: CBM_14), found in various molluscs and arthropods. Peritrophin-A is an extracellular matrix protein that contains six conserved cysteine residues that are predicted to form three disulphide bridges. Four of these cysteine positions are conserved in ML5H8. This observation indicates that carbohydrate-binding properties of the abalone calcified matrix proteins play probably an important role in the formation of the calcified shell.

### Function and evolution of shell matrix proteins

By comparing the largely unique biomineralising protein sequences we report here to those from other molluscs, we confirm the dramatic differences in gene sets used to build calcified shells [8-10]. Furthermore, our data illustrate the remarkable diversity of shell proteins within a *Haliotis *genus; except for Perlwapin [6], none of the peptides we detected displayed similarity to any of the dozen shell proteins described from *H. laevigata *or *H. rufescens *(for review see [11]). These data support the idea that shell matrix proteins are less evolutionary constrained than would be expected for closely related species [30]. This is unexpected given the similar nacre and prismatic shell microstructures of all abalone shells, and highlights the need for functional characterisation of these proteins.

### The value of EST libraries for biomineralisation focused proteomic analyses

This study highlights the value of EST libraries constructed from shell secreting tissues when used in conjunction with a shotgun proteomic approach. The efficiency of such a proteomics approach relies on the completeness of the EST data set. In this study, we exploited a pool of 3 different EST libraries generated from larval or adult calcifying tissues [8-10]. While some peptides were represented in all three EST libraries, others only appeared in one or two libraries (Table 1). This supports the previous observation of differential expression of biomineralising transcripts during development [9]. Additional fine scale variation in biomineralising gene expression is also likely to occur; recent investigations of the pearl oyster *Pinctada fucata *mantle have detected daily variation in the expression levels of shell protein-encoding genes [31-33]. These observations support the idea that the molecular mechanisms of molluscan shell formation follow finely regulated chronological events. This point should be carefully considered, especially when sampling tissues for transcriptomic analysis of extracellular calcifying matrices. We are aware that these EST data sets used in this study are not exhaustive, and future efforts will likely reveal additional shell proteins For example, a recent proteomic analysis of the calcified skeleton of the sea urchin *Paracentrotus purpuratus *revealed an unexpected diversity of matrix proteins, due to the availability of a draft genome [24,25,34].

## Conclusions

In the field of molluscan biomineralization, this work constitutes the first attempt to marry transcriptomic data with proteomic data in order to identify novel shell matrix proteins. By directly interrogating EST libraries with proteomic data generated from extracted shell matrices, we were able to annotate and describe 14 shell proteins from the nacreous and the prismatic layers, 12 of which are novel. Screening transcriptomic data with peptide sequences is therefore a powerful approach to annotate shell proteins and to fully identify their primary structure. The challenge that now faces the field is to characterise the function of the ever-growing list of novel biomineral associated proteins, using *in vivo *or *in vitro *techniques.

## Abbreviations

AIM: acid-insoluble matrix; ASM: acid-soluble matrix; SEM: scanning electron microscopy; WAP: whey acidic protein

## Competing interests

The authors declare that they have no competing interests.

## Authors' contributions

BM conceived the study, performed organic matrix extraction and sample preparation. AM and LD carried out the MS peptide analysis. BM, AM and FM performed the MS data analysis, did the data searches and the protein annotations. BM and FM participated in the design and the coordination to draft the manuscript. DJ and FM supplied methodological expertise and participated in the sequence analysis. DJ and BD supplied the shell sample and the EST dataset. All authors took part in the design of the study and were critically involved in data interpretation and manuscript drafting. All authors read and approved the final manuscript.

## Supplementary Material

Additional file 1 Table S1: Conceptually derived sequences and MS/MS observed peptides of shell matrix proteins of *Haliotis asinina*. Click here for file

Additional file 2 Table S2: List of shell matrix proteins of *Haliotis asinina *identified with only one unique matching peptide by MS/MS. Click here for file
