# Supplementary material for: Proteomic analysis of the organic matrix of the abalone Haliotis asinina calcified shell
Source: Proteome Sci. 2010 Nov 4;8:54. doi: 10.1186/1477-5956-8-54 (PMC2989941; doi:10.1186/1477-5956-8-54)
Supplement: Additional file 1 — Table S1: Conceptually derived sequences and MS/MS observed peptides of shell matrix proteins of Haliotis asinina. [file 1477-5956-8-54-S1.DOC]

**Additional file 1. List of shell matrix proteins of *Haliotis asinina* identified by MS/MS.**

| **Protein name [GenBank AN]** | **Transcript/protein lengths** | **Protein sequence + matching peptides**  **(Signal peptides; * = Stop codon)** | **Matching peptide sequences** | **Peptide MASCOT scores** |
| --- | --- | --- | --- | --- |
| ML5A7  [DW986289] | 909 bp / 244 aa | MVLHVITALLSIGLCYGMPPSGTTAAPTAYNGAAAQESLIPQQAHHARASLFPQQQRNPSYQTMGSYGSSLFDPSVFPVAHAPASQAGGLLGRAVARTSGGQANAGASSQNLMRSIMQNTMTGRLMGLDNQEIAHLNSVRTLGVGHATIHRLMKIDQIPSYNYYLALKNKPAQFSKAQNYLMTLNRMENHATDSQLEAMGARMLMQRNMDPDLARMVQLDAARGVYGDRLQRVLLGGSQMSLLG* | MVQLDAAR  NMDPDLAR  ASLFPQQQR  AQNYLMTLNR  SIMQNTMTGR  TLGVGHATIHR  IDQIPSYNYYLALK  LMGLDNQEIAHLNSVR  MENHATDSQLEAMGAR | 51  41  48  59  48  42  42  79  49 |
| HasCL10contig2  [EZ420619] | 1593 bp / 507 aa | MLRVPLLVLCLALSVGADYYGYGWGRNGGGGGSGGGSGSSRASASASASARANSIGNLVGRLTSLVDASASARASASANAGGFGGSGAGGSGGNGFGGGSGGSGFGGGSGGSGFGGGSGGSGFGGGSGGSGFGGGSGGSGFGGGSGGSGFGGASASASAQALASATAELQAAQDAYDQASAYAEATARAAANGGSLDSSALASAIASAEASVSARGASIIARARARAEATVRAARRSFASAQASAEASVSAVRSAEGRARSFARAVARARASARAAIAGVRSSGRAFASATARARASVSAAARAVARARAQAVARARASIRASASASARASASAAAEARAAAYARVQVAAAAAARAAASAASASASASASGSSFGSGGSGGSGNGGFGSFGASANAVANAFAQAFGGGLGNGGNGGNGNGGNGGNGGNGNGGNGGNGNGGNGGNGNGGNGGNGGNGNGGNGGNGNGGNGRNGNGGNGRNGNGGNGGNGNGRNGRGGRYYYGSSDYYY* | ANSIGNLVGR  ASVSAAAR  AFASATAR  ASASASASAR  ASASAAAEAR  VQVAAAAAAR  LTSLVDASASAR | 61  52  52  91  78  59  65 |
| P0012N13_463  [GT274423] | 463 bp / +126 aa | ?LFVGLTSAKYHDVCQLPRDPGPCRAYIPLYYFNSRTCLCEKFVYGGCQGNANRFDTVEDCRRRCGGGDLCSLPRDSGPCEAAIPRWWYNKRTNRCQRFTYGGCEGNANNFKTLDECRFQCRRRSTY* | TLDECR  WWYNK  FDTVEDCR  AYIPLYYFNSR  DSGPCEAAIPR  FVYGGCQGNANR | 31  31  53  69  77  101 |
| ML1E6  [DW986219] | 806 bp / 200 aa | MILQAALFLAGLTVVSGSICCPPKQFNSYQYVTFVNSTTTIRALYYIVYDGDNQRYLITGDRNNKQLVGTTKVIYDYKKRIAYSIDAKARTCSKFPVQGQFEDQENVCVPGGAEILGPLFYGYNQSRLNSQSYAYNTTSLDGSHHNVVTTVSEDDCVPIVICTITTGGPGGNSLYTVGYNDFYPGIKDITVFDIPPYCNA* | VIYDYK  YLITGDR  IAYSIDAK  QLVGTTK  DITVFDIPPYCNA  QLVGTTKVIYDYK  ALYYIVYDGDNQR | 32  60  53  31  36  58  77 |
| P0006O07_675  [GT272916] | 675 bp / +179 aa | ?PLGAATSNIPPQYARSTLQPTGLTSRAQSYPTNTNPGPSAKGNLVLPLNWQLLNSPASQIPTQSTTTFRSNPPLPPVVPGRRNTSPFFFPKPTRPLSFRQILDFLGRIRATKELDCKTVSEALSLNLPKFYYPLSCDDKCPPPSVCRHVGLVGFCCPPHVTDQLIWMVGLAERFKVLGG* | QILDFLGR  FYYPLSCDDK  STLQPTGLTSR  TVSEALSLNLPK  SNPPLPPVVPGR  GNLVLPLNWQLLNSPASQIPTQSTTTFR | 60  45  50  62  53  73 |
| P0025F23_658  [GT276990] | 658 bp / +204 aa | ?DDWNDNGNGDNGDDDDFWDDWDDDRFDDDRFDDDRFDDDRFDDDRWDDDDNDDWDDDDRWGDDDNDDWDDDDRWGDDDNDDWDDDDRWGDDDNDDWDDDDRWGDDNGNGNGNGNGNGNGDDDDDNGGYAFLRRALARASARARAAASAAGRSRGGSGGSGGSGGSGGSARARARARARAFASARASSGNGVNGGNGKKRSYTSY* | GGSGGSGGSGGSGGSGAR  WDDDDNDDWDDDDR  WGDDDNDDWDDDDR | 92  120  131 |
| ML3A11  [DW986237] | 553 bp / 172 aa | MVPVARASLFTLACLLVSVCAQNFPGPPPGAVLRGPFPPARPSLTNRVHPPTSAAGMQLMRRMAVSEAVLSSDYTKRMTALDAIQNIPCVFRNPVLKYLLMDYMDMKPLPLPGSVQSQMSPATQSMILTDRGRRLQAGKLLNSRCVQSHDRLGALMYDIAEVPMAASALAGR* | GPFPPARPSLTNR  MAVSEAVLSSDYTK  MTALDAIQNIPCVFR  VHPPTSAAGMQLMR | 32  128  89  87 |
| ML6A10  [DW986342] | 698 bp / 130 aa | MNSLIFLAVICSTAYVAFAQNTPIRPPFLPGPPAAGAPRIPAKGVAGLARQPQKSLLNNPYLMMMGDKYFEVAALDMLMDPSSQVSPIQQMVIAKSADLSPVDMMVANRLRGAQGTGMRSMYFPAILAMM* | GAQGTGMR  SMYFPAILAMM  SADLSPVDMMVANR  SLLNNPYLMMMGDK  YFEVAALDMLMDPSSQVSPIQQMVIAK | 40  52  81  65  82 |
| ML8B1  [DW986463] | 473 bp / 117 aa | MAAYCQAQGLDVVLAALLGAINQQPSRQQFQQQQQQQRQPQLQQQQQQQGIQQQPQGLQHQQQQFGLTQQHGQGRRQNIVQPNPASQNNNRMMLDMLLLNQIAQSNRMNTLAFIMAN* | MNTLAFIMAN  QQFQQQQQQQR  MMLDMLLLNQIAQSNR | 39  56  70 |
| P0011O14_517  [GT274178] | 517 bp / +162 aa | MWYKVLGIVSLCSVYVSTQGSNLQCKIAGGGELIPIRTPEYRAWVPLGEVGLNVSDSKVLIFAARSCFGAHILLQNNAEDFKNAVYEIVIGGMKNTRSGIRQCVGCKFDEWVKDKALNCRFFNYFWISWCGDEVWVGHGYRPPHNRILRYKYENQPVINAAS? | VLIFAAR  IAGGGELIPIR  NAVYEIVIGGMK | 45  85  49 |
| 6G3  [GD272908] | 848 bp / 201 aa | MILQVVLLLACLSGAIVSTGACCPPSRFNAFQYVTIVNSTTRTRGLYYMVYDGPNERYLLTGDRLKNLYGTTRVIYDYKKGIAYNIDVQKRSCTTFPLHGKFEDQENVCVPRDAVYTGRSAYGFDQGALHSWSYEYNRTHPDGRHQNIETTVTKENCIPIVTTTISTDASGGNSLHILGYNDFYPGIRDISMLEIPSYCRA* | YLLTGDR  GIAYNIDVQKR  GLYYMVYDGPNER | 60  63  50 |
| ML3E9  [DW986256] | 616 bp / 152 aa | ?LILCVVVCTAAVLGTAAGYESQLPGCPPGAYPAICARYCYSDRDCASGYYCCNTGCLNICVPKPKPGLCPSITQSPCRGNVCNNDQDCPGNRKCCGKPGCKRCYRPEKPGSCPARKYEAGPCVVYCDGDFDCPGDKKCCGGCPRLCEKPCYD* | CCGGCPR  LCEKPCYD  GNVCNNDQDCPGNR | 32  33  83 |
| ML5B8  [DW986296] | 755 bp / 209 aa | MGYFPYLAVFVCLLASGDAQWKGLRGSTKASWVRVVSPTLNVTQEAYIWDADSGISFISRSKLLTGTSINRNRAYTDYNQPKIAIKFRKDVGRTCILLDVASALIGTFNETSTNLQALTVLDTTEEKSYQSVGTVLSATSQSAFDLQHPFIQKKCSDRNYNYLATTELTAGAAPPPASDKFTVFTTWGKVHLYILQSTGLVITTTTEAP* | FTVFTTWGK  NYNYLATTELTAGAAPPPASDK | 48  77 |
| ML5H8  [DW986339] | 418 bp / 110 aa | MKYLVGCLCLAAICLSAGAQRKKVKCNLYMLKRSLVHYISRDRTGTRVMPCPPGTIFSEGHCGCVSLTNGCDMRPYSGRKYKHWIHGKWRTRYCPAGTTLNQSKCVCDHA* | CNLYMLK  SLVHYISR  YCPAGTTLNQSK | 34  32  50 |
|  |  |  |  |  |
