# Supplementary material for: Proteomic analysis of the organic matrix of the abalone Haliotis asinina calcified shell
Source: Proteome Sci. 2010 Nov 4;8:54. doi: 10.1186/1477-5956-8-54 (PMC2989941; doi:10.1186/1477-5956-8-54)
Supplement: Additional file 2 — Table S2: List of shell matrix proteins of Haliotis asinina identified with only one unique matching peptide by MS/MS. [file 1477-5956-8-54-S2.DOC]

**Additional file 2. List of shell matrix proteins of *Haliotis asinina* identified with only one unique matching peptide by MS/MS.**

| **Protein name [GenBank AN]**  **BLAST** | **Transcript/protein lengths** | **Protein sequence + matching peptides**  **(Signal peptides; * = Stop codon)** | **Matching peptide sequences** | **Peptide MASCOT scores** |
| --- | --- | --- | --- | --- |
| ML6F9  **[DW986386]**  **Brichos domains** | 810 bp / 205 aa | MKMLLATGIAILFLAYAACQLQRNEEGTRVTYKWQRNINPEAEPFYVEEHASILRRGVILMSSESSALHEKQYHRSQSLHDFTKGILAIKHLDSRGGKGICFLTHPAKTFRETVEDLQKRTEKGKVVLEEPEHFYRLSSKEPVPRTDSISVNRFCSGAPMYQVEEFYRPFPRDYPWIIRTLCIYWPLRFWWPLDFPGDFNPIYYV* | GVILMSSESSALHEK | 89 |
| CL226  **[DW986253]**  **Unknown** | 801 bp / 92 aa | MWYKVLGIVSLCSVYVSTQGSNLQCKIAGGGELIPIRTPEYRAWVPLGEVGLNVSDSKVLIFAARSCFGAHFYFRTMRRTSRMLFMRLSLEE* | IAGGGELIPIR | 60 |
| ML3D4  **[DW986253]** Antimicrobial | 279 bp / 93 aa | MTFLLFILLLPLVTCTWPGLWPEYNHEDDCLDVAIIGAGIGGTYTGYRLRNRGLKLAIFEYSDRIGGRMFTAQIPTVPGTTVDFGAMRVDPQK? | LAIFEYSDR | 32 |
|  |  |  |  |  |
